# Fire risk and safety for people living with dementia at home: A narrative review of international literature and case study of fire and rescue services in England

**DOI:** 10.1177/14713012251320251

**Published:** 2025-02-13

**Authors:** Tiffeny James, Andrew Clark

**Affiliations:** The Institute for Lifecourse Development, 4918 Univeristy of Greenwich, UK

**Keywords:** dementia, fire risk, fire and rescue services, assistive technology, safety at home

## Abstract

**Background:** Most people living with dementia prefer to continue living at home. However, as dementia progresses, people may become more susceptible to risk including cooking accidents that can lead to fire. This is a common concern cited by people living with dementia, family carers, and healthcare professionals, but research in this area is lacking. **Methods:** To identify initiatives, interventions, and guidance around fire safety for people living with dementia at home, first we conducted a narrative review of international literature. Next, we used England as a case study by searching all English fire and rescue services websites. We also sent Freedom of Information requests to all services to explore what information is held about fire incidents involving people living with dementia in England. **Findings:** Eight peer-reviewed articles were eligible for inclusion. Existing literature suggests that assistive technologies such as stove shut-off devices can be difficult for people living with dementia to use and cause additional problems and risks. All English fire services offer ‘Home Fire Safety Visits’, designed to help those vulnerable to fire identify and reduce risk at home however, only four specify that people living with dementia are eligible. Eleven services and two UK dementia charities have produced fire safety guidance for people living with dementia in England. Dementia awareness training in one fire service increased support offered to people living with dementia including provision of assistive technologies. Fire services in England do not record dementia status routinely and methodological issues mean that available data is unlikely to be accurate. **Conclusions:** There is scope for developing standardised dementia fire safety guidance and awareness training. Further research is needed to explore what types of assistive technologies people affected by dementia want and would find acceptable. We conclude with suggestions for fire safety policy, practice, and future research for England and internationally.

## Introduction

Most people living with dementia prefer to continue living at home (Førsund et al., 2018; Lord et al., 2016), and enabling them to do so is key priority for research and policy both in England (Department of Health., 2015; James Lind Alliance, 2013) and internationally (World Health Organisation, 2017). Over time, however, changes in cognitive function including to memory, thinking, attention, and executive function can make people living with dementia at home more susceptible to risk (Backhouse et al., 2022). This includes risk of fire associated with the misuse of appliances and cooking accidents which is frequently raised as a concern by family carers (James, 2022; Jang et al., 2023; Stevenson & Taylor, 2018; Thoma-Lürken et al., 2018) and health and social care professionals supporting people living with dementia (Backhouse & Ruston, 2022; Taylor et al., 2018). A common accident described in practice is fire caused by the use of a plastic kettle on a gas hob by a person living with dementia (Alzheimer’s Society, 2015; Caldwell et al., 2014; Heward & Kelly, 2020; James, 2022). Family carers have cited this as the reason for their relative with dementia moving into residential care (Caldwell et al., 2014) or the family’s home (James, 2022).

As well as increased risk of causing accidental fire, dementia can affect a person’s ability to recognise and respond to fire (Alzheimer’s Society, 2015). Exit routes may be forgotten or inaccessible if, for example, the door is locked from the inside and keys are misplaced. People living with dementia may no longer understand the sound of an alarm or what to do when they hear one, and changes to olfaction can impact the ability to smell smoke or gas. Whilst families and paid carers can help mitigate risk of fire for people living with dementia, they are unlikely to be able to provide 24-h supervision. This is especially true for the approximately 120,000 people living with dementia who live alone in England - a number which is set to double by 2039 (Alzheimer’s Society, 2019). Risk of injury from fire may be unintentionally heightened by carers locking doors from the outside in an attempt to reduce risks associated with leaving the home such as falling or become lost (Evans et al., 2016; Morgan, 2009).

Data on fires involving people living with dementia is lacking. In England, for example, the Home Office do not record or report on the number of fires involving people living with dementia, despite the heightened risk for this population. What we do know is that people over the age of 65 are most at risk of serious injury or death from fire compared to other age groups (Home Office, 2024). In the year ending March 2023, 47% of all fire-related fatalities in England were in people aged 65 years and over. This was a similar proportion to the previous year (49%) (Home Office, 2023). Whilst not wanting to conflate ageing and dementia, these statistics are relevant as most people living with dementia are aged 65 and older, and the risk of developing dementia roughly doubles with every five-year increase in age (Prince et al., 2014). In 2023, the non-fatal casualty rate from fires was highest in those aged 80 years and over (183 casualties per million people). Despite the known links between ageing, dementia, and heightened risk of injury from fire, the evidence for understanding and responding to these risks remains low.

Among the few studies that have reported on fire risk in the context of dementia, there are data collection and reporting limitations and mixed results, and studies do not account for “near-misses” or fires that do not result in hospitalisation. Cohort studies suggest that people living with dementia are more likely to be hospitalised due to burns (Chen et al., 2018) and more likely to die from thermal injuries (Bicknell et al., 2020) than people without dementia. However, these studies do not specify the cause of burns/thermal injury which could be due to fire, water, steam, or hot appliances. In studies looking at burns from fire specifically, people living with dementia were more likely to have been burnt from ignition of clothing than people without dementia (Harvey et al., 2016) but overall, were equally (Harvey et al., 2016) or less (Meuleners & Hobday, 2017) likely to be admitted to hospital for fire-related burns than people without dementia. This could be because people living with dementia stop using appliances that can lead to fire, such as the stove, due to cognitive impairment or are prevented from using such appliances by families or professionals due to safety concerns (Doughty & Orton, 2014).

People living with dementia and family carers are generally tolerant of risk when the benefits of an activity outweigh the potential risks (Hoe et al., 2023), but arguably not when it comes to fire (Backhouse & Ruston, 2022). Healthcare professionals have overestimated risk of fire for people living with dementia by at least half (Taylor et al., 2018) and when evaluating risk, report being more concerned by the severity of a potential risk rather than likelihood of it occurring (Stevenson & Taylor, 2017). Healthcare professionals are more likely to adopt risk-averse approaches to meet organisational requirement or due to a lack of time, rather than supporting people to manage risk (Stevenson et al., 2019; Stevenson & Taylor, 2017, 2018). A risk-averse approach might be removing the stove and replacing it with a microwave (Doughty & Orton, 2014; Taylor et al., 2018) rather than supporting a person to use the stove or installing technologies to help. Assistive technologies have the potential to help people living with dementia remain safe and independent at home by, for example, aiding memory, supporting activities of daily living, and lowering the risk of falls (Brims & Oliver, 2019). Some technologies have the potential to reduce fire risk and harm from fire such as stove shut-off devices which can stop the gas or electricity supply after a certain period of time or when a certain temperature is reached (Taylor et al., 2018).

In England, the Fire and Rescue National Framework stipulates that fire and rescue services must make provision for promoting fire safety, including fire prevention (Home Office, 2018). Fire and rescue services are expected to target their fire safety, prevention, and protection resources on those who are most vulnerable to fire, which includes people living with dementia. Research around fire risk reduction for people living with dementia, however, is lacking. A 2022 review of risk and risk mitigation for people living with dementia and their homecare workers identified 27 studies, of which only two mentioned fire (Backhouse et al., 2022). In a separate review of risk assessment for people living with dementia, nine of the 20 included studies mentioned fire risk, but none focussed on this directly (Hoe et al., 2023). This paper aims to explore existing literature and knowledge around fire risk and fire safety for people living with dementia at home to provide an overview of:

1. any guidance, initiatives, or interventions in place to prevent and protect people living with dementia from fire in England and internationally;
2. the information held by fire and rescue services about fire incidents involving people living with dementia in England;
3. any gaps in knowledge, research, and practice around fire risk and safety for people living with dementia.

## Methods

We conducted an integrative narrative review of existing literature and knowledge around fire risk and safety for people living with dementia at home (Whittemore & Knafl, 2005). This method is well suited when exploring under-researched topics and when including literature with diverse methods (Sukhera, 2022). We searched Applied Social Sciences Index and Abstracts (ASSIA), Cumulative Index to Nursing & Allied Health (CINAHL), Social Science and Policy, and Web of Science for the terms “dementia OR Alzheimer* AND fire”. We included national and international literature published in English between 2000 and February 8th, 2024, when the searches were conducted. To be included, results had to describe guidance, initiatives, or interventions around fire safety for people living with dementia in the community i.e., not in residential care or nursing homes. We excluded conference abstracts and reviews, but sought out relevant papers from these and checked reference lists of relevant papers.

Next, we narrowed the focus to England. Based on experience in both research (Clark et al., 2015; Clark & Smith, 2018) and practice, the authors were aware of the work that many fire and rescue services in England have implemented to promote safety and reduce fire risk for people living with dementia in the community. To supplement the international literature review, we explored the breadth of such initiatives in England to highlight examples of good practice that can be shared with fire and rescue services in other countries. To do this, we searched Google for the phrase “dementia fire England” and screened the results until they were no longer relevant. Next, we used the search function in the websites of all 43 English fire and rescue services to search for the word “dementia” (see Appendix 1 for details of fire and rescue services and their websites). We collected information about any guidance, initiatives, or interventions around dementia and fire safety including the provision of home fire safety visits (HFSVs) for people living with dementia. HFSVs involve a visit to persons home to assess them, their home, and any behaviours within the home that may increase the risk of fire. Visits are carried out by fire fighters, fire prevention specialists, or in some cases, specific community or home fire safety officers. HFSVs are typically targeted towards those who are more vulnerable to fire, and are designed to help people reduce and prevent fires in the home; identify potential fire risks in the home; and create an escape plan. Fire and rescue service professionals provide guidance on how to make homes safer from fire risk by, for example, testing fire alarms and addressing hazards. Fire and rescue services can provide fire alarms, and in some cases, fire-retardant bedding. They may also recommend and sometimes provide assistive technology.

Lastly, to explore the information held by fire and rescue services about fire incidents involving people living with dementia in England, we contacted all 43 English fire and rescue services and requested the following information for the years 2021, 2022, and 2023:

i. the number of fires they attended involving people living with dementia;
ii. the number of false alarms involving people living with dementia; and
iii. the number of fire-related fatalities involving people living with dementia.

We requested this information under the Freedom of Information Act 2000. Freedom of Information legislation exists in over 80 countries and supports individuals’ right to request access to information held by various government bodies (Walby & Larsen, 2012). It has been described as an underused but “powerful tool for social researchers” (Savage & Hyde, 2012, p. 303). Research which gathers data using Freedom of Information legislation does not require ethical regulation or approvals because the procedures involved in Freedom of Information requests must uphold ethical values of anonymity, confidentiality, and protection from harm (Walby & Luscombe, 2018).

## Findings

### Initiatives, interventions, and guidance around fire safety for people living with dementia

**Figure 1.**
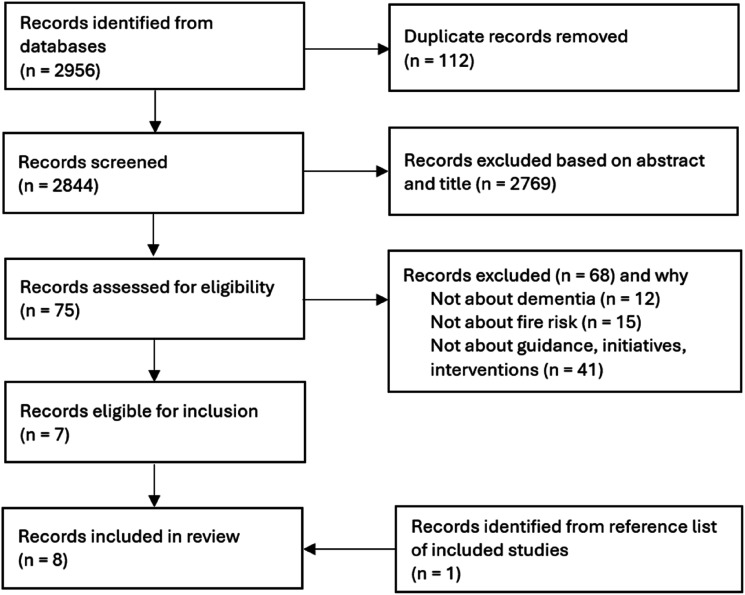
Flow diagram showing study section process.

Searches on scientific databases yielded 2844 results after deduplication. After reviewing titles and abstracts, 75 were identified for full text screening and of these, seven were eligible for inclusion. We identified one additional study (Nygård, 2009) from the reference list of an included study bringing the total to eight (Figure 1 shows the study selection process). The majority (6/8, 75%) of included studies were qualitative (Appel et al., 2020; Cahill et al., 2007; Holthe et al., 2018; Malmgren Fänge et al., 2020; Nygård, 2009; Starkhammar & Nygård, 2008). There was also one case file audit (Nygård et al., 2008) and one project evaluation (Heward & Kelly, 2020). Characteristics and main findings of included studies are in Table 1. We identified an additional 13 relevant documents or webpages from the grey literature and fire and rescue service website searches. These were all guidance around fire safety and dementia. From the fire and rescue service website searches, we found that all 43 English fire and rescue services offer HFSVs for eligible people. We report our findings in three parts: 1) assistive technology; 2) improving knowledge to reduce risk; and 3) home fire safety visits.

**Table 1.** Characteristics and main findings of included studies.

Author and year	Aims and objectives	Methods	Participants	Findings related to fire
Appel et al. (2020)	To test the usability of “SafeHome” app, a game developed to teach safety strategies in the kitchen for people living with dementia	Questionnaire of user satisfaction (*n* = 13) and one semi-structured focus group (*n* = 8)	Family carers of people living with dementia (*n* = 13)	Participants found it useful to have potential kitchen risks highlighted, and felt that the game was an entertaining method of learning, helping you to “remember longer”
Cahill et al. (2007)	To explore whether technologies (including a stove shut-off device) could be used and were considered useful by people living with dementia and their family carers	Mixed methods using semi-structured interviews and descriptive statistics	People living with dementia (*n* = 20) and family carers (*n* = 20)	Three participants trialled the stove shut-off device, but all withdrew before the end of the study due to major technical issues (causing a gas leak and cutting off mains supply); or problems adjusting to the device (changing the cooker’s appearance making the person living with dementia reluctant to use it)
Malmgren Fänge et al. (2019)	To explore the experiences, needs, and benefits of using sensor-based technology (including a smoke alarm that automatically alerts family members when activated) for safety and independence in people living with dementia	Semi-structured interviews	People living with dementia (*n* = 9) and family carers (*n* = 21)	Generally, family carers found technology helpful, especially when other systems (e.g., normal smoke alarms) did not work or the person living with dementia did not hear them. For some, being the first point of call for automatic alarms impacted personal relationships i.e. moving them from being a relative to a carer
Heward and Kelly (2020)	To report on outcomes and outputs of an evaluation of a collaborative project between a university and fire and rescue service in England to develop knowledge of fire risks and safety strategies in the homes of people with dementia, and share this with fire and rescue service staff and volunteers	Project evaluation	Fire and rescue service staff and volunteers (*n* = 76)	Fire and rescue service staff and volunteers reported increased dementia knowledge after receiving training. Support provided by the fire and rescue service to people living with dementia increased e.g. provision of fire-retardant bedding and referrals to other services, and the fire and rescue service had started developing safety guidance for people affected by dementia
			Post-training dementia knowledge and awareness: Immediately post training (*n* = 74); 6-months post training (*n* = 5)	
Holthe et al. (2018)	To examine experiences of family carers using assistive technology (including a stove shut-off device) as a means of supporting people with young onset-dementia	Repeated semi-structured interviews	Family carers of people with young-onset dementia (*n* = 13)	Family carers reported issues with stove shut-off devices including “annoying” beeping after use and the installation of the device cutting power to the electric stove
Nygård (2009)	To explore the views of professionals involved in providing stove shut-off devices for people with dementia and cognitive impairment, and how their views guided their actions	Interview focus groups (*n* = 5)	Professionals (*n* = 23) including: Occupational Therapists, nurses, homecare professionals, home modification officers, and an Electrician across 5 groups: 1 (*n* = 5); 2 (*n* = 7); 3 (*n* = 5); 4 (*n* = 3); 5 (*n* = 3)	Professionals felt that the purpose of the device is to provide safety, rather than support people living with dementia to continue cooking. When introducing such devices, they took into account the motivation of the person living with dementia and the timing of introduction which could risk being “too soon” or “too late”
Starkhammar and Nygård (2008)	To explore experiences of people living with dementia and family carers using a stove shut-off device	Interviews and observations	People living with dementia (*n* = 9) and family carers (*n* = 5)	Participants reported both benefits e.g. the person living with dementia being able to remain or restart cooking on the stove; and challenges with using the device. Many found it difficult to use e.g. not knowing how to override the shut-off for longer cooking times, and some found that it created more issues and confusion e.g. by cutting off the stove part way through cooking something
Starkhammer and Nygård (2009)	To identify the characteristics of people provided with stove shut-off devices and investigate the application procedures of the devices	Audit of home modification service case files	Case files (*n* = 938) of people provided with a stove shut-off device	Of the 939 people provided with a stove shut-off device, 788 (84%) experienced problems associated with dementia or undiagnosed cognitive impairment, and 769 (83%) of all people were living alone. People without cognitive impairment were more likely to choose to have the auditory alarm to alert them when the stove was about to be cut off compared to people with cognitive impairment

#### Assistive technology

The majority of identified studies (6/8, 75%) were about assistive technology for people living with dementia. Three of these discussed a range of assistive technologies for people living with dementia including at least one device designed to prevent or reduce the risk of fire: two with stove shut-off devices (Cahill et al., 2007; Holthe et al., 2018) and one with a smoke alarm that automatically alerts family members once activated (Malmgren Fänge et al., 2020). Three further studies focused on stove shut-off devices only (Nygård, 2009; Nygård et al., 2008; Starkhammar & Nygård, 2008). From the fire and rescue service website searches, we identified three fire and rescue services that recommended stove shut-off devices specifically for people living with dementia (Cambridgeshire Fire & Rescue Service, Greater Manchester Fire & Rescue Service, and London Fire Brigade). London Fire Brigade have produced an extensive assistive technology catalogue with a specific section relevant for people living with dementia (London Fire Brigade, 2015).

Whilst assistive technology could provide a sense of safety for some people living with dementia and family carers (Starkhammar & Nygård, 2008), it also created challenges and confusion. Stove shut-off devices caused a gas leak (Cahill et al., 2007) and blown fuses causing the mains electricity to short (Cahill et al., 2007; Holthe et al., 2018; Nygård, 2009; Starkhammar & Nygård, 2008), leaving some people to eat meals cold when they could not get the electricity back on (Nygård, 2009). Some experienced beeping from devices designed to alert users to risk, as “annoying” but felt they “just have to live with it” to prevent fire (Holthe et al., 2018, p. 759). There was sometimes a lack of knowledge from family members (Starkhammar & Nygård, 2008) and professionals (Nygård, 2009) around when or why the device would switch the stove off i.e., whether it was after a certain amount of time or after recaching a certain temperature. On average, devices were programmed to switch the stove off after 30 minutes (Nygård et al., 2008) however, this caused issues when cooking meals that required a longer time. One person living with dementia described using an egg timer to remind them to turn the stove back on again (Starkhammar & Nygård, 2008). Switching the timer off before the 30 minutes was also challenging as this required additional steps (Starkhammar & Nygård, 2008).

Assistive technologies for people living with dementia need to be introduced at the “right time” (Holthe et al., 2018; Malmgren Fänge et al., 2020; Nygård, 2009; Starkhammar & Nygård, 2008). Stove shut-off devices, when introduced too late, were viewed as “an odd artefact that obstructed cooking” (Starkhammar & Nygård, 2008, p. 187) and stopped people from using the stove at all due to its changed and unfamiliar appearance (Cahill et al., 2007). Stove shut-off devices, for example, must be introduced whilst the person living with dementia is still capable of using the stove, which was not always the case according to professionals (Nygård, 2009) and demonstrated by a family carer who said: “This assistive technology seems very nice, but it is too late for us to use this now. The dementia has progressed too far.” (Holthe et al., 2018, p. 757). As well as introducing technologies too late, professionals highlighted the risk of introducing items too soon which could cause people living with dementia to feel “infringed” when their capacity to manage everyday devices, such as a stove, is questioned (Nygård, 2009, p. 58).

People living with dementia need the capacity to understand, learn about, and develop daily habits around new technologies for them to be successfully incorporated into everyday life (Holthe et al., 2018; Malmgren Fänge et al., 2020; Nygård, 2009; Starkhammar & Nygård, 2008). Successful implementation requires time, engagement, and willingness by family carers (Malmgren Fänge et al., 2020; Nygård, 2009; Starkhammar & Nygård, 2008). Family carers had left written instructions, practiced using the device with the person living with dementia, and even stayed with their relative for the few weeks following installation to help them become familiar with it (Starkhammar & Nygård, 2008). Some family carers, however, doubted whether the instructions would be remembered after they left (Starkhammar & Nygård, 2008). Some homecare professionals viewed it as “almost impossible” for clients with dementia to learn how the stove shut-off device worked (Nygård, 2009, p. 60). Some technologies, such as a smoke alarm that automatically alerts family members when activated, require little to no teaching or learning (Malmgren Fänge et al., 2020). Whilst most family carers valued being alerted to an adverse event such as fire, others did not wish to be responsible for their relative’s everyday safety, preferring to maintain the role of relative rather than carer (Malmgren Fänge et al., 2020). People discussed wanting to receive information about assistive technology “at the right time” (Holthe et al., 2018, p. 757), but opinions about when the right time was varied across studies.

Family carers were interested in assistive technologies that could help the person living with dementia maintain meaningful and safe activities, especially when alone (Holthe et al., 2018) however, it is not clear if this includes using the stove to cook. Professionals in one study discussed the stove shut-off device as a “life saver”, allowing continued engagement in activities such as cooking or heating meals safely and enabling people living with dementia to remain living at home for longer (Nygård, 2009, p. 61). Most, however, felt that the device was used as a fire precaution suggesting that it “only existed in the homes of those who, for safety reasons, should no longer use their stoves and who only did so by mistake” (p. 61). In these cases, the device was sometimes perceived as a substitute and therefore barrier to people receiving the care they really needed, which might include a move into supported housing (Nygård, 2009).

#### Increasing risk reduction knowledge

We identified several initiatives designed improve knowledge about dementia and/or fire risk associated with dementia and how to reduce this. Initiatives to increase knowledge were aimed at people living with dementia, family carers, health and social care professionals supporting people living with dementia, and fire and rescue service professionals.

##### Educating fire and rescue services about dementia

One study reported outcomes from a collaborative project between a university and fire and rescue service in England (Heward & Kelly, 2020). An education programme that included information about dementia was developed to inform understanding of fire risks associated with dementia. This was implemented in one fire and rescue service using a “train the trainer” model. The authors report an increase in staff knowledge and awareness about dementia immediately after taking part in the training (*n* = 76). After six months, respondents (*n* = 5) said they strongly agreed (*n* = 4, 80%) or agreed (*n* = 1, 20%) that they felt more aware of the signs and symptoms of dementia. The evaluation also reported an increase in the support offered to people living with dementia by the fire and rescue service including provision of assistive technologies and fire-retardant bedding. At the time of the evaluation, the fire and rescue service was in the process of developing a range of fire prevention guidance for people living with dementia and their family carers.

We did not collect data about dementia training for fire and rescue services. However, we note that several fire and rescue services reported providing training for their staff and volunteers. Information about staff training was not presented consistently and tended to be reported in news articles either produced by the fire and rescue service or local newspapers. It was not possible to systematically collect data on the number of services who had or were continuing to provide dementia training, or the number of staff who had received training or information about dementia. Some fire and rescue services that reported providing training referred to the Alzheimer’s Society’s “Dementia Friends” dementia information sessions (Alzheimer’s Society, 2023). This 60-min session provides a basic overview of dementia, but is not specific to fire safety and the dangers that people living with dementia may face.

##### “SafeHome” app

One study reported on the acceptability and feasibility of an app-based game for family carers of people living with dementia (Appel et al., 2020). To use the game, users had to identify potential hazards in a virtual kitchen which, when clicked on, produced a text box with suggestions on how to reduce the risk of that hazard. Participants found it useful to have potential kitchen risks highlighted, and felt that the game was an entertaining method of learning, leading you to “remember longer”.

##### Fire safety guidance

We identified 13 documents/websites from England with practical tips and guidance around fire safety for people living with dementia. The majority of these were from fire and rescue services (*n* = 11) and two were from dementia charities in England: the Alzheimer’s Society (n.d.) and Dementia UK (2023). Most other fire and rescue services had some general fire safety information on their websites, but made no reference to people living with dementia, symptoms of dementia, or those who may be more vulnerable to fire. Fire safety guidance by dementia charities was included in their general guidance for safety at home for people living with dementia. Both charity guides recommend that people living with dementia contact their local fire and rescue service for a HFSV.

Eleven out of 43 (26%) fire and rescue services had some form of written guidance around fire safety relevant for people living with dementia on their websites. These were: Cumbria Fire & Rescue Service; Derbyshire Fire & Rescue Service; Devon and Somerset Fire & Rescue Service; Dorset and Wiltshire Fire & Rescue Service; East Sussex Fire & Rescue Service; Essex County Fire & Rescue Service; Kent Fire & Rescue Service; Lancashire Fire & Rescue Service; London Fire Brigade; Northamptonshire Fire & Rescue Service; and Staffordshire Fire & Rescue Service. Of these, eight had produced guidance specific to dementia; two referred to people who are more vulnerable to fire including people living with dementia; and one did not mention dementia explicitly, but highlighted factors associated with dementia that may make a person more vulnerable to fire such as reduced cognitive function or capacity to understand what to do in the event of a fire. Three were directed at family or paid carers of people living with dementia, and the rest were for general audiences including people living with dementia, families, and professionals. Guidance from four fire and rescue services was particularly detailed: East Sussex Fire & Rescue Service; Essex County Fire & Rescue Service; Derbyshire Fire & Rescue Service; and Staffordshire Fire & Rescue Service. Additionally, four services were offering information sessions for health and social care professionals supporting people who may be more vulnerable to fire, though only one service website specified that this could include people living with dementia.

#### Home fire safety visits (HFSVs)

According to their websites, all 43 fire and rescue services offered HFSVs, though one service had paused their HFSV service as of February 2024 due to high demand. Fire and rescue service websites varied in terms of their description of eligibility for a HFSV but broadly, eligible persons were those who either: smoke; are over the age of 65; or have a disability or long-term health condition which may affect their ability to respond to and escape from fire. Four out of 43 (9%) fire and rescue service websites specified that people living with dementia are eligible for a HFSV; three mentioned cognitive impairment but not dementia specifically; 11 listed older age and disability generally; and the remaining 25 did not specify eligibility and/or required people to complete an online form to determine their eligibility. Eighteen fire and rescue services also offered a “Safe and Well” service as part of their HFSVs. This involves a more holistic assessment of the person’s health and well-being around, for example, their mobility, weight, mental health, alcohol use, and feelings of loneliness and isolation (see Kingstone et al., 2022 for more information). Visiting professionals can provide advice, signpost people to other services, and refer to other local services with the person’s consent.

### Information held about fire incidents involving people living with dementia in England

All 43 fire and rescue services responded to our Freedom of Information requests. Different services provided different amounts of information. Nearly half of fire and rescue services (21/43; 49%) provided information for at least one query. Table 2 shows the number of services that provided information for each query and Table 3 shows the number of different types of incidents reported in the years 2021, 2022, and 2023 by services that provided information. Some services provided one number for the three-year period rather than breaking down the number of incidents for each year. In these cases, we divided the number provided by three and spread this across the three years.

**Table 2.** Number of fire and rescue services out of 43 that provided data for each query.

	Fire and rescue services that provided data *n* (%)
Fires involving people living with dementia	20 (47)
False alarms involving people living with dementia	16 (37)
Fatalities from fire-related incidents involving people living with dementia	12 (30)

**Table 3.** Number of fire-related incidents, false alarms, and fatalities reported for people living with dementia in the years 2021, 2022, and 2023 by fire and rescue service that provided information.

	2021	2022	2023	Total
Fires (out of 20 services)	92.9	95.9	73.9	262.7
False alarms (out of 16 services)	348.6	382.2	354	1084.8
Fatalities (out of 12 services)	1.6	0.6	1.6	4

The main reason given for not providing information was that the information was not recorded (*n* = 19 services). Fire and rescue services advised that any information about health conditions such as dementia would only be recorded narratively within the free text of incident reports or within information about “human factors” related to the cause of fire. One service indicated that their reporting system was not designed in a way to search for such information, and another advised that the amount of time required to provide this information exceeded the amount of time services are required by law to spend on a Freedom of Information request. Other services were able to search the free text for the word “dementia” but specified that data may not be accurate due to a range of methodological issues. We were advised, for example, that fire officers are not required to record dementia status routinely; that dementia status would only be recorded if it was pertinent to the fire; and that fire officers are not expected to reliably gauge whether someone has dementia meaning not all incidents involving people living with dementia may be recorded as such. Two services reported that the low number of fire-related fatalities for people living with dementia may make the individuals involved identifiable, breaching the Data Protection Act 2018 and principles of the Freedom of Information Act 2000 and therefore did not provide this information.

#### Fires, false alarms, and fatalities

Across the 21 services that provided data on fire incidents involving people living with dementia, the total number of incidents reported for the three-year period was 262.7. The number of incidents reported by individual services for the three-year period ranged from 0 to 78. The highest number of incidents reported by an individual service within a one-year period was 31.

The total number of false alarms reported across 16 services for the three-year period was 1084.8. This figure ranged from 0 to 229 within individual services. The highest number reported by an individual service within a one-year period was 91. Three services provided a breakdown of the type of false alarms such as being due to device malfunction or accidental activation; good intent; or malicious intent. There was one incident reported of a false alarm with malicious intent. Twelve services provided data about fire-related fatalities involving people living with dementia. Across the 3-year period, four fatalities were reported from four different services.

This wide range in the number of fire incidents and false alarms reported by individual fire and rescue services is likely because the size of areas covered by individual fire and rescue services also varies greatly, indicated by the number of fire stations within each service. For services that provided information to us, the number of fire stations ranged from 5 to 102.

## Discussion

This paper provides an overview of what is known about fire risk for people living with dementia and what exists to prevent and protect people living with dementia at home from fire. Most of our findings are based on fire and rescue services in England however, the implications and recommendations are relevant for other countries. In reviewing international peer-reviewed literature, we found that interventions to reduce fire risk for people living with dementia typically describe assistive technologies. Such technologies were reported as being confusing to use; difficult to incorporate into everyday life; and causing additional challenges for people living with dementia. Around a quarter of English fire and rescue services have produced fire safety guidance for people living with dementia and/or vulnerable populations, some of which recommend assistive technologies. All fire and rescue services in England provide Home Fire Safety Visits (HFSVs) which offer a practical approach to identifying and reducing fire risk for people living with dementia however, only 4/43 (9%) services specified that people living with dementia are eligible. Staff and volunteers in some English fire and rescue services had received information or training about dementia, but this was not reported consistently. We found that fire and rescue services in England do not record dementia status routinely and where it is recorded, methodological issues and lack of standardised reporting mean that available data is unlikely to be accurate.

Our review has identified examples of good practice around dementia and fire safety in England, as well as areas for improvement. All English fire and rescue services offer HFSVs, for example, but few specify that people living with dementia are eligible, instead referring to “vulnerable groups”. People living with dementia may not recognise themselves as vulnerable or as having dementia. To address this, we recommend that all fire and rescue services offering HFSVs specify that people living with dementia are eligible, and describe some common examples of fire incidents involving people living with dementia such as burning pans or getting distracted and forgetting that the stove is on (Evans et al., 2016). This can help members of the public recognise when they are eligible for a HFSV, even without a formal dementia diagnosis.

It is clear that fire and rescue services encounter a number of individuals with, or attend incidents where dementia is present (Heward & Kelly, 2020). Providing dementia training to fire and rescue service staff and volunteers, especially to those who do HFSVs could improve their ability to identify cognitive impairment; respond to the associated fire risks; and in the case of “Safe and Well” visits, refer consenting individuals on for further investigation and support if appropriate. In studies exploring the potential for HFSVs to support help-seeking for mental ill-health, fire and rescue service staff identified training needs around communication and coping skills to avoid being impacted by difficult and distressing experiences (Fisher et al., 2023, 2024) which could also be relevant for dementia. Future research could explore what, if any, dementia training fire professionals receive, where the gaps are, and what else can be done by, for example, exploring whether fire and rescue services would want and benefit from the development of a standardised dementia training to be rolled out to all services as part of induction training both in England and internationally. Such training should include an understanding of dementia in relation to other determinants of fire risk including co-morbidities, individual mobility, household overcrowding, and housing type as well as other behaviours that can increase fire risk such as smoking or alcohol consumption (Clark et al., 2015). Providing dementia training to fire and rescue service staff and volunteers could also improve their ability to identify cognitive impairment and therefore record it in their systems. Recording would also likely be improved by having a systematic way to record dementia status however, this does not guarantee that information will be accurate as fire and rescue staff and volunteers are not expected to reliably gauge whether someone has dementia, and would remain dependent on householders revealing a diagnosis.

Whilst assistive technologies may play a role in reducing fire risk for people living with dementia, for technologies to be adopted, they must provide an improvement in everyday life and be easy to handle as indicated in previous research (Lindqvist et al., 2013), and be introduced at the “right time” as identified in our review. Though assistive technologies could provide a sense of safety for some people living with dementia (Cahill et al., 2007; Starkhammar & Nygård, 2008), they could also cause additional problems, echoing work with the wider population (Clark & Smith, 2018). A randomised controlled trial of assistive technologies found that they did not help people living with dementia to remain living at home for longer (Gathercole et al., 2021). They may actually prevent people from getting the care they need which would help them live better (Nygård, 2009). Further work is needed to explore what types of assistive technologies people living with dementia and their families want, what they would find acceptable to reduce fire risk, and exactly when and in what circumstances such technologies should be introduced. Future design of interventions must include people affected by dementia to ensure their acceptability and usability (Hill et al., 2024).

### Strengths and limitations of this review

This paper presents, to our knowledge, the first review of published and grey literature about domestic fire risk and fire safety in the context of dementia. Our findings suggest that this is an under researched area. However, as we only searched for the words “dementia” and “fire”, it is possible that relevant papers were not identified in the literature review if they discussed fire risk by using words like “smoke” or “gas”, or by referring only to appliances that can cause fire such as “stove” or “oven”. Whilst we only included English fire and rescue services in our exploration, our review provides a framework for researchers to assess the status of dementia and fire safety in other countries. We have identified areas of good practice and provided recommendations which are applicable to fire and rescue services and people living with dementia in all countries including recommendations that fire and rescue services undertake dementia training; offer HFSVs; and produce fire safety guidance for people living with dementia. Due to the lack of standardised recording of dementia status by fire and rescue services, our findings cannot offer an accurate picture of the number of fire incidents and false alarms involving people living with dementia in England. Similarly, these findings do not tell us anything about “near misses”, such as when a fire occurs but is extinguished by a person living with dementia, their relatives, neighbours, care workers, or other persons without fire and rescue service being called. Exploring this would likely require surveys with family carers, which could be the focus of future research.

### Conclusion

The key messages from this review are that there has been little focus on fire safety for people living with dementia in research thus far, and the information available from fire and rescue services in England is not currently sufficient to accurately describe the risk of fire for people living with dementia at home. There is a need to strengthen the evidence base around risk and experiences of fire among individuals affected by dementia so that services can be better aligned to address that risk and provide support. Our review indicates the ongoing need for dementia awareness raising and training among fire and rescue service personnel including among those providing community safety advice. We close, though, with a caveat to our discussion. It is important to avoid labelling those living with dementia who experience domestic fires as helpless and/or vulnerable victims. This is particularly important when targeting households and individuals for fire safety advice and preventative measures. Our intention in writing this paper is not to suggest that fire risk is a reason to automatically deny people living with dementia the right to remain in their own home, but to highlight the important role that fire and rescue services have in enabling independence and safety at home for people living with dementia.

## Appendix

### Appendix 1: List of all 43 English fire and rescue services and their links to their websites

**Table:**

Fire and rescue service	Website
Avon fire & rescue service	https://www.avonfire.gov.uk/
Bedfordshire and Luton fire & rescue service	https://www.bedsfire.com/
Royal Berkshire fire & rescue service	https://www.rbfrs.co.uk/
Buckinghamshire fire & rescue service	https://www.bucksfire.gov.uk/
Cambridgeshire fire & rescue service	https://www.cambsfire.gov.uk/
Cheshire fire & rescue service	https://www.cheshirefire.gov.uk/
Cleveland Fire & rescue service	https://www.clevelandfire.gov.uk/
Cornwall fire & rescue service	https://www.cornwall.gov.uk/
Cumbria fire & rescue service	https://www.cumbriafire.gov.uk/
Derbyshire fire & rescue service	https://www.derbys-fire.gov.uk/
Devon and Somerset fire & rescue service	https://www.dsfire.gov.uk/
Dorset and Wiltshire fire & rescue service	https://www.dwfire.org.uk/
County Durham and Darlington fire & rescue service	https://www.ddfire.gov.uk/
East Sussex fire & rescue service	https://www.esfrs.org/
Essex county fire & rescue service	https://www.essex-fire.gov.uk/
Gloucestershire fire & rescue service	https://www.glosfire.gov.uk/
Greater Manchester fire & rescue service	https://www.manchesterfire.gov.uk/
Hampshire fire & rescue	https://www.hantsfire.gov.uk/
Hereford and Worcester fire & rescue service	https://www.hwfire.org.uk/
Hertfordshire fire & rescue service	https://www.hertsdirect.org/fire
Humberside fire & rescue service	https://www.humbersidefire.gov.uk/
Isles of Scilly fire & rescue service	https://www.scilly.gov.uk/community-safety/fire-rescue
Kent fire & rescue service	https://www.kent.fire-uk.org/
Lancashire fire & rescue service	https://www.lancsfirerescue.org.uk/
Leicestershire fire & rescue service	https://www.leicestershire-fire.gov.uk/
Lincolnshire fire & rescue service	https://www.lincolnshire.gov.uk/LFR
London fire brigade	https://www.london-fire.gov.uk/
Merseyside fire & rescue service	https://www.merseyfire.gov.uk/
Norfolk fire & rescue service	https://www.norfolk.gov.uk/safety/norfolk-fire-and-rescue-service
North Yorkshire fire & rescue service	https://www.northyorksfire.gov.uk/
Northamptonshire fire & rescue service	https://www.northantsfire.gov.uk/
Nottinghamshire fire & rescue service	https://www.notts-fire.gov.uk/
Oxfordshire fire & rescue service	https://www.oxfordshire.gov.uk/fire_service
Shropshire fire & rescue service	https://www.shropshirefire.gov.uk/
South Yorkshire fire & rescue service	https://www.syfire.gov.uk/
Staffordshire fire & rescue service	https://www.staffordshirefire.gov.uk/
Suffolk fire & rescue service	https://www.suffolk.gov.uk/suffolk-fire-and-rescue-service/
Surrey fire & rescue service	https://www.surreycc.gov.uk/people-and-community/fire-and-rescue
Tyne and wear fire & rescue service	https://www.twfire.gov.uk/
Warwickshire fire & rescue service	https://www.warwickshire.gov.uk/fireandrescue
West midlands fire & rescue service	https://www.wmfs.net/
West Sussex fire & rescue service	https://www.westsussex.gov.uk/fire-emergencies-and-crime/west-sussex-fire-and-rescue-service/
West Yorkshire fire & rescue service	https://www.westyorksfire.gov.uk/
